# A Review of Microbial Decontamination of Cereals by Non-Thermal Plasma

**DOI:** 10.3390/foods10122927

**Published:** 2021-11-26

**Authors:** Vladimír Scholtz, Jana Jirešová, Božena Šerá, Jaroslav Julák

**Affiliations:** 1 Department of Physics and Measurements, University of Chemistry and Technology, Prague, Technická 5, 166 28 Prague, Czech Republic; vladimir.scholtz@vscht.cz; 2 Department of Environmental Ecology and Landscape Management, Faculty of Natural Sciences, Comenius University in Bratislava, Ilkovičova 6, 842 15 Bratislava, Slovakia; bozena.sera@uniba.sk; 3 Institute of Immunology and Microbiology, First Faculty of Medicine, Charles University and General University Hospital in Prague, Studničkova 7, 128 00 Prague, Czech Republic; jaroslav.julak@lf1.cuni.cz

**Keywords:** electrical discharge, active particles, food contamination, decontamination

## Abstract

Cereals, an important food for humans and animals, may carry microbial contamination undesirable to the consumer or to the next generation of plants. Currently, non-thermal plasma (NTP) is often considered a new and safe microbicidal agent without or with very low adverse side effects. NTP is a partially or fully ionized gas at room temperature, typically generated by various electric discharges and rich in reactive particles. This review summarizes the effects of NTP on various types of cereals and products. NTP has undisputed beneficial effects with high potential for future practical use in decontamination and disinfection.

## 1. Introduction

A cereal is any ‘grass’ grown for its fruit called grain (botanical term: caryopsis). Cereals include crops, such as wheat, rice, corn, barley, rye, oats and millet, that underpin the world’s food supply [1]. The word cereal comes from ‘Ceres’, the Roman goddess of agriculture. Cereals were first cultivated by ancient Sumerians in the area of the Middle East between the Euphrates and Tigris rivers. Cereal grains provide more food energy content than any other type of crop. Cereal grains in their unprocessed form are called ‘whole grains’ and are a rich source of carbohydrates, fats, oils, protein, vitamins and minerals [1,2,3].

Grain contamination can occur in the field or during harvest and storage. Bacteria that may be present on the grain before harvest are mainly *Staphylococcus*, *Enterococcus*, *Enterobacter*, *Pseudomonas*, *Xanthomonas*, *Alcaligenes*, *Flavobacterium*, *Bacillus* or *Clostridium*. Cereal grains and cereals are often also infested with fungi of the genera *Fusarium*, *Alternaria* (typical field fungi), *Aspergillus* and *Penicillium* (typical warehouse fungi).

From the point of view of microbial quality control, the most important process is immediately after the harvest and thus in the storage of grain. In order to prevent the undesirable development of microorganisms, it is advisable to store the grain at a temperature from 10 to 15 °C and at a relative humidity of maximum 75%.

Due to the importance of cereals as food for humans and animals, interest in this area is still growing. Non-thermal plasma treatment of grains is one of the new fields in studies of surface changes, influencing the rate of germination, disinfection of carcasses or influencing the quality of cereal products at present. The use of non-thermal plasma for the microbial decontamination of grains before sowing or grains as food is probably very promising and may be applicable in practice. Moreover, the new term “plasma agriculture” was also considered [4]. This was the impetus for our decision to write a review article on this topic.

In the storage of food and especially cereals, microorganisms are considered unwanted carriers of infections either for the consumer or for the next generation of plants [5]. Some microorganisms can also produce various toxins, e.g., aflatoxin [6]; the production of large quantities of other mycotoxins is also common. Bacterial botulinic neurotoxin may also occur infrequently [7]. The mycotoxins most usually associated with cereal grains are ochratoxins, deoxynivalenol, zearalenone and fumonisins too [8].

Several methods have been developed to eliminate these undesirable agents. The various food processes having effects on mycotoxins include cleaning, milling, brewing, cooking, baking, frying, roasting, flaking, alkaline cooking, nixtamalization, extrusion and thermal treatment [8,9,10]. In addition to chemical agents, they may also be based on the action of various physical phenomena, above all by thermal treatment. Most of the food processes have variable effects on mycotoxins, with those that utilize high temperatures having the greatest effects. In general, the processes reduce mycotoxin concentrations significantly but do not eliminate them completely [8]. Among methods, the non-thermal plasma (NTP) has been proved to be an effective way to keep the mentioned agents under control. The use of non-thermal plasma as a flexible sanitizing method was described in the early reviews of Misra et al. [11] and Niemira et al. [12]. Chizoba-Ekezie et al. [13] reviewed specific areas of NTP applications, including microbial decontamination of food products, packaging material processing, modification of the functionality of food materials, dissipation of agrochemical residues and others. This paper also provides a summary of plasma chemistry and sources, factors influencing plasma efficiency and strategies to enhance the effect of NTP. Various methods of non-thermal food processing, namely pulsed electric field, pulsed light, ultraviolet radiation, high-pressure processing, ozone treatment, ionizing radiation, ultrasound and, last but not least, non-thermal plasma, were recently reviewed by Chacha et al. [14]. Selected methods of wheat and wheat products were also mentioned there. Some applications of plasma treatment and related methods were also included in another recent review by Domonkos et al. [15]. Concerning the possible side effects of plasma, several papers summarized in the review [16] mentioned no or minimal impacts on the physical, chemical, nutritional and sensory attributes of various products.

NTP is a partially or fully ionized gas which is not in local thermodynamic equilibrium, meaning that the temperature of free electrons is high (typically over approximately 10,000 K), while the temperature of hard particles (molecules and ions) and therefore the overall temperature remains low. Due to the specific mechanism of plasma generation, this temperature may remain at the room temperature or increase up to thousands of K. However, due to low heat capacity of plasma, it may not heat up the object on which it is applied. NTP is typically generated in various electric discharges and rich in reactive particles; see, e.g., [7,17,18,19,20,21,22,23,24]. For seeds or grain treatment, it is typically generated in an air atmosphere or in an atmosphere containing O_2_ and N_2_, where the so-called reactive oxygen and nitrogen species (RONS), such as O, ^1^O_2_, O^2−^, OH^−^, OH^.^ and NO_x_, arise. Detailed explanations of processes and effects on microorganisms have been described in many reviews, e.g., [25,26]. This non-thermal physical method is gradually attracting attention as a potential application in many areas of the agricultural and food industry; see, e.g., [27,28,29,30,31,32]. Among other positive effects, such as accelerated germination that leads in selected cases to a more resilient population and consequently higher yields [33,34], NTP is also able to inactivate not only most species of microorganisms [35,36], but also toxins, especially mycotoxins [37,38,39,40]. Some works even describe the effect of NTP on pest control, as mentioned in Kaur et al. [41].

Recently, the phenomenon of so-called plasma-activated water or plasma-treated water has also been described, where NTP is applied only to pure water or other medium [42,43,44]. Active particles mediate the desired properties, although they accumulate to a lesser extent than in direct action. PAW is often referred to as a microbicidal agent; for example, the inactivation of bacteria on strawberries can be mentioned [45]. Unfortunately, we did not find any work dealing with the action of PAW in cereals.

This review summarizes the microbicidal effects of NTP studied on various types of cereals and cereal products as important parts of human nutrition. It is intentionally designed to provide an overview of all the beneficial properties of NTP achieved without highlighting the nature of NTP generation, which is often complex and diverse. The purpose of the review is to give an overview of the significant features and not the details, the inclusion of which would prolong and obscure the whole text. For any details, the kind reader is sure to find the relevant original works.

## 2. Microbicidal Effects of NTP on Cereals

The works cited are arranged in paragraphs according to cereal type. An overview of the links found is given in Table 1.

### 2.1. Wheat

The largest share of studies deal with this crop. Wheat is an important, tradable commodity. Its use is versatile, from direct feeding to animals, through the production of flour, to the production of ethanol. Microbiological protection of wheat grain is important both in the field and in grain processing.

Thomas-Popo et al. [46] reported the inactivation of both artificial and natural contamination of wheat grains. For artificial contamination by *E. coli* and *Salmonella enterica*, the total cfu decreased for the initial cca 7 log_10_ by 3–4 log_10_ after 20 min of plasma treatment. For natural contamination, the decrease in total cfu of mesophiles, psychrotrophs and *Enterobacteriaceae* after 20 min of treatment was almost 1, more than 2 and 1.4 log_10_, respectively. On the contrary, the yeast and molds were completely destroyed after only 10 min.

The following two related works [47,48] reported the inactivation of bacterial endospores of *Bacillus amyloliquefaciens* and *Geobacillus stearothermophilus* in wheat grains. While in the first case, the total cfu of *B. amyloliquefaciens* was reduced by 2 log_10_ from initial 10^6^ cfu/g after 30 s, using the other source of NTP in the second case led to the 0.8 log_10_ and 3 log_10_ after 5 min and 60 min, respectively.

According to Zahoranova et al. [60], the concentration of epiphytic bacteria decreased from the initial cca 5 × 10^4^ cfu/g by more than 1 log_10_ after 600 s. Epiphytic yeast was not detected and filamentous fungi were completely inactivated from the initial 600 cfu after 120 s of treatment. For artificial contaminations, the less resistant *Fusarium nivale* and *F. culmorum* were completely inhibited after 90 s, *Trichothecium roseum* after 180 s and *Aspergillus flavus* after 240 s; however, the most resistant, *A. clavatus*, was not totally inhibited after 300 s.

Selcuk et al. [49] used *Aspergillus paraciticus* (corresponding to *parasiticus*) and *Penicillium* spp. isolated from foods for artificial contamination in 5 × 10^6^ cfu/g of grains and reported a reduction of more than 2 log_10_ after 30 min of treatment.

Hoppanova et al. [50] treated the grains inoculated with *Fusarium culmorum* spores in a concentration of 10^5^ g grain^–1^ with plasma or in combination with 10% of Vitavax2000 fungicide. Complete inactivation occurred after 180 s and 60 s of plasma exposure alone and plasma exposure with fungicide, respectively.

Filatova et al. [51] used artificial contamination with *Fusarium culmorum* and natural contamination with *Alternaria* spp.; the infection levels decreased from 40% to 7% and from 4% to 2%, respectively. Inactivation of these fungi led to better germination, growth and grain yield.

In [52], the authors did not report the inactivation of fungal spores, but the resistant behavior of the treated samples to fungus attack, which decreased from 40% to 20% after 2 or 4 min of treatment.

In the work of Los et al. [53], the authors inactivated the natural microflora of mesophilic bacteria, yeasts and molds of 10^4^–10^5^ cfu/g. Maximal reductions of 1.5 log_10_ CFU/g for bacteria and 2.5 log_10_ CFU/g for fungi were achieved after 20 min of treatment. The following study [54] demonstrated that direct plasma exposure for 20 min significantly reduced the concentration of all pathogens. The reduction levels for the vegetative cells of *Bacillus atrophaeus* were higher than for all the fungal species tested, while the spores of *B. atrophaeus* were the most resistant. Repeating sublethal plasma treatment did not induce resistance to ACP in either *B. atrophaeus* or *A. flavus* spores.

Kordas et al., 2015 [55], reported the decrease in fungal contamination on grains from an initial cca 250 cfu per 100 grains to cca 25 cfu per 100 grains after 10 s of treatment.

Works related to wheat are the most numerous and show the possible applications of plasma in the widest range; as for the inactivation of the microorganism, so for increasing the resistance of crops. So far, all works are on a more or less laboratory scale.

Insects may also cause serious problems. The following four papers described the possible inactivation of *Tribolium* and other species in wheat by NTP.

Shahrzad et al. [56] reported the killing of *Tribolium confusum* and *Ephestia kuehniella* larvae in wheat from cca 300 to 0 in 20 s. Ratish Ramanan et al. [57] achieved the total elimination of eggs, larvae and adults of *T. castaneum* in wheat flour containing 10 eggs, 5 larvae or 5 adults in 15 min. In [58], 25 insects of *T. castaneum* and *T. confusum* per 30 g of wheat showed 100% mortality after 15 min of exposure. On the other hand, a very low mortality of *T. castaneum* of approximately 5% in wheat grains was reported in an otherwise chaotic paper [59].

### 2.2. Rice

Rice grains are often attacked by various microbiological pathogens [72,73]. Rice, as one of the most consumed cereals in the world, was the focus of the several following papers.

The first attempts were reported by Kang et al. [64], who treated with NTP rice grains infected by *Fusarium fujikuroi* mold spores that cause bakanae disease. They sprayed the spore suspension of 10^6^ cfu/mL on rice plants. The harvested grains were then exposed to NTP, which caused the number of infected grains to decrease from 100% of the control set to 20% in grains exposed for 30 min.

The follow-up study [61] reported the successful effect of NTP on the control of two rice seed-borne diseases. It also examines the bakanae disease caused by *Fusarium fujikuroi* mold and the blight disease caused by *Burkholderia plantarii* bacteria. The bakanae disease severity index and the percentage of plants with symptoms were reduced to 18% and 8% after 10 min of exposure. The index of blight disease was reduced to 39%.

Natural rice contamination was also studied in the following two papers. Park et al. [65] reported the decontamination of natural contamination of brown rice grains by bacteria, yeasts and molds and reported a reduction of more than 1.5 log_10_ after 10 min of exposure. In [66], complete inactivation of natural contaminants (pathogenic fungi and other microorganisms) in a rice grain husk after 1 min of exposure was reported.

Inactivation of artificial contamination by *Aspergillus oryzae*, *Penicillium digitatum* spores and *E. coli* (initial concentrations of contaminants are not given) was reported in [62]. The surface of rice and lemons was sterilized after 20 min of irradiation with a combination of plasma and UV light.

Finally, an attempt to industrial application was reported in [63], where the development of a large-scale NTP generator followed by a UV-C treatment was described. To evaluate the efficacy of rice natural microorganisms decontamination, the number of natural bacteria was reduced from initial 5.6 log_10_ to 1 log_10_ cfu/g; for yeasts and molds, the reduction was from 3.7 log_10_ to 2 log_10_ cfu/g after 7 min of treatment.

Works related to rice present similar results as those for wheat; however, the attempt to industrially up-scale gives hope for further development and usage.

### 2.3. Maize

Maize is currently grown all over the world, with the United States being one of the world’s largest producers. Several papers devoted to corn decontamination start with Selcuk et al. [49], who used the *Aspergillus parasiticus* and *Penicillium* spp. food isolated for artificial contamination of 5 × 10^6^ cfu/g of grains. They reported an approximate 70% reduction after 30 min of treatment. The paper [67] focused mainly on grain germination but also reported that, after 4 min of grain treatment, the inhibition of artificial contamination of grains by *Fusarium verticillioides* and *F. graminearum* was achieved so that all grains, contrary to the control, germinated without visible mold growth occurrence.

In [51], the authors used artificial contamination of *Fusarium culmorum* and natural contamination of *Alternaria* spp. The infection level decreased slightly from 76% to 66% and from 30% to 10%, respectively. This inactivation of fungi caused by grain treatment led to better germination, growth and grain yield.

In [68], the authors investigated the inhibition of the native microbiota and potentially dangerous pathogens (*Aspergillus flavus*, *Alternaria alternata* and *Fusarium culmorum*) in grains. Complete devitalization of the native microbiota was observed after 60 s of treatment for bacteria and 180 s for filamentous fungi. For artificial contaminations, total elimination from the initial 3–4 log_10_ (CFU/g) was observed after 60 s for *F. culmorum* and after 300 s for *A. flavus* and *A. alternata*.

In [74], the decrease in artificial infection with *A. flavus* and *A. parasiticus* from the initial 10^7^ cfu/g by 5 log_10_ in 5 min was reported. The natural contamination of the fungi of the initial almost 10^4^ cfu/g and of the aerobic mesophilic bacteria of the initial 10^3^ cfu/g was totally inactivated after 3 min. Much lower inhibition was reported in [69], where the initial number of more than 200 fungi per 100 grains was reduced to 30% after 20 min of treatment.

Although all cited works are devoted to the fungi only, it can be assumed that, for other microorganisms, the decontamination efficiency will be comparable to previous crops.

### 2.4. Barley

It is one of the oldest cereals in the world and is geographically widespread. Today, most barley grown, especially winter barley, is used for feed purposes. Barley is an important feed grain for many countries, especially for those that are not suitable for maize production. Barley also received attention for NTP decontamination.

In [70], the concentration of artificial contamination with *Aspergillus niger* and *Penicillium verrucosum* in the total mold count of more than 5 log spores/g grains was reduced by 2.5–3 log. Furthermore, the use of air plasma also resulted in a decrease in ochratoxin A concentration from 56 (untreated) to 20 ng/g after 3 min.

The two previously mentioned works also deal with barley. Selcuk et al. [49] used *Aspergillus parasiticus* and *Penicillium* spp. isolated from foods for artificial contamination at 5.006 cfu/g of grains and reported a reduction of more than 1 log_10_ after 30 min of treatment. Hoppanová et al. [50] treated grains inoculated with *Fusarium culmorum* spores in a concentration of 10^5^ g grain^−1^ with plasma or in combination with 10% Vitavax2000 fungicide. Complete inactivation occurred after 120 s and 60 s of plasma exposure alone and plasma exposure with fungicide, respectively.

The paper [67] is focused mainly on the germination of grains. They reported inhibition of artificial contamination of grains by *Fusarium verticillioides* and *F. graminearum* after 4 min of treatment, insomuch as all grains germinated without visible mold growth as opposed to the control. In the work [53], the authors inactivated both native microflora and artificial contamination. For the natural microflora of mesophilic bacteria, yeasts and molds of 10^4^–10^5^ cfu/g, maximum reductions of 1.5 log_10_ CFU/g for bacteria and 2.5 log_10_ CFU/g for fungi were achieved after 20 min of treatment. For artificial contamination, a total reduction of more than 3 log_10_ was observed after 20 min of exposure for *E. coli*, *Bacillus atrophaeus* vegetative cells and *Penicillium verrucosum* spores, while the reduction for the endospores of *B. atrophaeus* reached only 2.4 log_10_ CFU/g.

Much weaker inhibition was reported in [69], where the initial number of more than 200 fungi per 100 grains was reduced by up to 20% after 20 min of treatment.

In the work [71], unusual plasma-processed air (PPA) was used for inactivation of *B. atrophaeus* (DSM 675) endospores on barley grains, where gas flows from the active plasma to the incubation bottles. The number of spores was reduced from the initial concentration of ~10^6^ CFU/per 10 g by 3.00 ± 0.33 log_10_ after 3 min of exposure.

Obtained results are again comparable with other crops, but the last cited work suggests the possibility of using PPA, which could markedly simplify the whole operating process and the transformation to real processing.

### 2.5. Miscellaneous

The following paper [49], devoted also to oat and rye, used the *Aspergillus parasiticus* and *Penicillium* spp. food isolated for artificial contamination. The initial concentration of 5 × 10^6^ cfu/g of grains was reduced by more than 1 log_10_ after 30 min of treatment for oat and approximately by 80% for rye.

## 3. Discussion

As can be seen from the previous lines, the microbicidal effects of NTP have been studied on a large number of different cereals. This has made it possible to produce this review, which shows, through a number of studies, that the application of NTP has great potential as a protection against harmful unwanted microorganisms.

NTP is a broad term that encompasses a number of differently arranged devices for its generation with specific geometrical and electrical arrangements and different sizes, allowing laboratories to pilot applications. There are many laboratories dealing with NTP in the world, which unfortunately also leads to the use of many different NTP sources and hardly comparable results. This fact is often also pointed out in other works but, so far, it does not seem to be remedied. The works of Shaw et al. [75] and Khun et al. [76] can be considered as one attempt to at least partially unify the methodology, where the authors tried to create a defined protocol enabling this comparison. Unfortunately, these works went unnoticed. This is also the main reason why we did not focus in this paper on the precise description of the NTP sources but more or less only on the presentation of possible NTP effects and applications. We are of the opinion that these applications can be achieved using any of the cited sources; the question remains regarding the degree of efficiency that can be further optimized.

From a biological point of view, there is obvious interest in studying the effects of NTP on a large number of microorganisms, which represent a major burden in the agriculture and food industry. Therefore, its effectiveness on the vast majority of known microorganisms such as bacteria and fungi can be inferred. These results vary for different apparatuses and microorganisms, as well as cereals, but it can be seen that the time required to significantly reduce the microbial load ranges from tens of seconds to tens of minutes. In the case of rice, the development of industrial equipment has already begun. If this or other similar equipment could be put into operation, it would represent a significant advance in food safety and, moreover, in a very ecological way in terms of the use of chemicals and energy consumption.

Finally, we express the hope that a successful implementation would significantly increase the prestige of the whole physics of non-thermal plasma, which has been intensively studying the microbicidal effects for almost two decades. However, for practical applications of the reported literature and mostly laboratory findings and possibilities, they will need to be developed to an operational scale applicable to large-scale applications. The one up-scaled system reported in [58] showed the real interest of food industry; however, it pointed to the new evidence in unexplored aspects. Hence, the toxicological safety should be investigated in more detail and also further research to enhance the microbial inactivation efficacy is assumed; in comparison to the laboratory level, the pneumatic conveyor systems for uniform treatment is proposed. The efficiency is also closely related to the energy consumption and needs to be included in consideration.

In our opinion, it would also be necessary to address the issue of biofilms. This issue has been discussed in a number of previous papers, e.g., [77,78]; they suggest that the involvement of biofilms can significantly affect laboratory findings found on planktonic cultures.

## 4. Conclusions

This review provides an overview list of cereals, namely wheat, rice, maize, barley and partly oats and rye, for which research on the microbicidal capacity of NTP has been carried out. It has been confirmed that NTP has an effect on grain surface contamination caused by bacteria (*Bacillus amyloliquefaciens*, *B. atrophaeus*, *Burkholderia plantarii*, *Escherichia coli*, *Geobacillus stearothermophilus*, *Salmonella enterica*) and fungi (*Alternaria alternata*, *Aspergillus clavatus*, *A. flavus*, *A. niger*, *A. orhyzae*, *A. parasiticus*, *Fusarium culmorum*, *F. graminearum*, *F. fujikuroi*, *F. nivale*, *F. poae*, *F. verticillioides*, *F. culmorum*, *Gibberella zeae*, *Mucor hiemalis*, *Penicillium* MS1982, *P. citrinum*, *P. chrysogenum*, *P. digitatum*, *P. verrucosum*, *Rhizopus stolonifer*, *Trichoderma sp.*, *Trichothecium roseum*) and by natural microbial contamination (without determination). Disinsection of the germs of various insect species is also processed for these cereal species. The previous lines give an overview of the possible beneficial effects of NTP for the microbial decontamination of cereal grains; some articles even described the possible inactivation of insects. We did not find any work that mentions a significant decline in cereal quality. NTP has the potential for practical use for decontamination and disinfection. So far, the first case may be the work on rice, where a large-scale apparatus for practical use was already constructed.

## Figures and Tables

**Table 1:** The effect of NTP on naturally and artificially introduced microorganisms on cereal seeds.

Plant		Pathogen Name (and Source)	Plasma Apparatus	References
Common Wheat	*Triticum aestivum* L.	bacteria—*Escherichia coli*, *Salmonella enterica* and natural microflora	dielectric barrier discharge system (60 Hz, 44 kV, 56.5 W, air)	[46]
		bacteria—artificially contaminated *Geobacillus stearothermophilus* and its endospores	atmospheric pressure dielectric barrier discharge (argon as a working gas, 8 kV, 10 kHz, or pulse frequency 5–15 kHz, pulse voltage 6–10 kV, Ar)	[47]
		bacteria—artificially deposited *Bacillus amyloliquefaciens* endospores	low pressure plasma circulating fluidized bed reactor (13.56 MHz, 8–12.8 mbar, oxygen gas admixture)	[48]
		fungi—artificial inoculation with *Aspergillus parasiticus* 798, *Penicillum* MS1982	low pressure cold plasma prototype unit (1 kHz, 20 kV, 500 mTorr, 300 W, air or SF_6_)	[49]
		fungi—*Fusarium culmorum*-artificial	diffuse coplanar surface barrier discharge (14 kHz, 20 kV, 400 W, air)	[50]
		fungi—artificial inoculation with *Fusarium culmorum* + natural contamination *Alternaria* sp. and *Fusarium* sp.	planar geometry capacitively coupled plasma reactor (5.28 MHz, 200 Pa, 0.025 W cm^−3^, air)	[51]
		fungi (native microflora)	low pressure argon plasma produced by plasma-enhanced chemical vapor deposition (600–850 V)	[52]
		native microflora; artificial—bacteria—*Escherichia coli*, *Bacillus atrophaeus var. niger*, *fungi-Penicillium verrucosum*	dielectric barrier discharge closed system (80 kV, 50 Hz, air)	[53]
		native microflora *Aspergillus candidus*, *A. flavus and Penicillium chrysogenum;* artificial—bacteria—*Escherichia coli*, *Bacillus atrophaeus*, fungi—*Penicillium verrucosum*, *P. citrinum*, *Aspergillus niger*	dielectric barrier discharge closed system (80 kV, 50 Hz, air)	[54]
		fungi—natural contamination—*Alternaria alternata*, *Alternaria botrytis*, *Aspergillus brasiliensis*, *Epicoccum nigrum*, *Fusarium culmorum*, *Fusarium poae*, *Gibberella zeae*, *Mucor hiemalis*, *Penicillium* sp., *Rhizopus stolonifer*, *Trichoderma* sp.	reactor with a packed bed (8 kV, 100 Hz–83 kHz, air)	[55]
		insecta—*Tribolium confusum*, *Ephestia kuehniella*	dielectric barrier discharge device (10 kV, 13 kHz, air)	[56]
		insecta—*Tribolium castaneum*	dielectric barrier discharge (1–10 kV, 50 Hz)	[57]
		insecta—*Tribolium castaneum* Herbst and *Tribolium confusum* Jacquelin du Val.	stationary pressure plasma jet based on a dielectric barrier discharge (13.56 MHz, 90–130 W, argon, oxygen/argon, nitrogen/argon mixtures)	[58]
		insecta—*Tribolium Castaneum*	cold plasma (argon, 800 V)	[59]
	cv. Eva	fungi—artificial—*Fusarium nivale*, *Fusarium culmorum*, *Trichothecium roseum*, *Aspergillus flavus* and *Aspergillus clavatus*, natural microflora	diffuse coplanar surface barrier discharge (14 kHz, 20 kV, 400 W, air)	[60]
Rice	*Oryza sativa* L.	inoculation with fungi—*Fusarium fujikuroi* isolate Ka52 (MAFF244851) and spores of *Fusarium fujikuroi* (collected by suspending the mycelial mat), bacteria—*Burkholderia plantarii*	atmospheric plasma apparatus—inductively coupled plasma (20 kV, c. 10 kHz, air)	[61]
		fungi—*Aspergillus oryzae* and *Penicillium digitatum* varieties (mold spores), bacteria—*Escherichia coli*	active oxygen species produced by the combination of atmospheric plasma (7–10 kV, 10 kHz) and UV light in ambient air	[62]
		natural mesophilic aerobic bacteria and yeast and molds of rice germ	large-scale plasma jet-pulsed light-ultraviolet (UV)-C system (2 kW, 1 kV, 30 Hz, air)	[63]
	var. *Hopyeong*	fungi—*Fusarium fujikuroi*	ozone and arc discharge plasma (10–15 kV, 3 Hz, water)	[64]
	used term: brown rice	native microflora—aerobic bacteria, yeasts and molds	corona discharge plasma jet under atmospheric pressure conditions (20 kV DC, 1.5 A, air)	[65]
	var. *Indica* cv. KDML105	seed-borne fungi	dielectric barrier discharge (~ 14 kVpp, ~700 Hz, air + Ar)	[66]
Maize	*Zea mays* ssp. *mays*	fungi—artificial inoculation with *Aspergillus parasiticus* 798, *Penicillum* MS1982	low pressure cold plasma prototype unit (1 kHz, 20 kV, 500 mTorr, 300 W, air or SF_6_)	[49]
		fungi—*Fusarium culmorum* and the natural contamination	planar geometry capacitively coupled plasma reactor (5.28 MHz, 200 Pa, 0.025 W cm^−3^, air)	[51]
		fungi—*Aspergillus flavus* and *Aspergillus parasiticus* spores + native microflora	atmospheric pressure plasma jet (5–10 kV, 18–25 kHz, max. 855 W, air and nitrogen)	[63]
		fungi—*Fusarium graminearum* and *Fusarium verticillioides* conidial spore	afterglow of a surface-wave microwave discharge (25 W, 2–8 mbar, Ar-O_2_, N_2_-O_2_)	[67]
	cv. Ronaldinio	fungi—*Aspergillus flavus*, *Alternaria alternata* and *Fusarium culmorum* and native mikrobiota	diffuse coplanar surface barrier discharge (14 kHz, 20 kV, 80 W cm^−3^, air)	[68]
	var. *Everta*	seed-borne fungi	glow discharge plasma (15 Pa, 200 W, air)	[69]
Barley	*Hordeum vulgare* L.	fungi—artificial inoculation with *Aspergillus parasiticus* 798, *Penicillum* MS1982	low pressure cold plasma prototype unit (1 kHz, 20 kV, 500 mTorr, 300 W, air or SF_6_)	[49]
		fungi—*Fusarium culmorum*—artificial	diffuse coplanar surface barrier discharge (14 kHz, 20 kV, 400 W, air)	[50]
		native microflora, artificial—bacteria—*Escherichia coli*, *Bacillus atrophaeus var. niger*, *fungi*—*Penicillium verrucosum*	dielectric barrier discharge closed system (80 kV, 50 Hz, air)	[53]
		fungi—*Fusarium graminearum* and *Fusarium verticillioides* conidial spore	afterglow of a surface-wave microwave discharge (25 W, 2–8 mbar, Ar-O_2_, N_2_-O_2_)	[67]
		seed-borne fungi	glow discharge plasma (15 Pa, 100 W, air)	[69]
		fungi—*Aspergillus niger* and *Penicillium verrucosum*	diffuse coplanar surface barrier discharge (15 kHz, 20 kV, 350 W, air, CO_2_, CO_2_ + O_2_)	[70]
		bacteria—*Bacillus atrophaeus* (DSM 675) spores	plasma-processed air generated by microwave discharge (2.45 GHz, 4 kW, air)	[71]
Rye	*Secale cereale* L.	fungi—artificial inoculation with *Aspergillus parasiticus* 798, *Penicillum* MS1982	low pressure cold plasma prototype unit (1 kHz, 20 kV, 500 mTorr, 300 W, air or SF_6_)	[49]
Oat	*Avena sativa* L.	fungi—artificial inoculation with *Aspergillus parasiticus* 798, *Penicillum* MS1982	low pressure cold plasma prototype unit (1 kHz, 20 kV, 500 mTorr, 300 W, air or SF_6_)	[49]

## Data Availability

Data sharing not applicable.
